# Assessing the relationship between sexual health literacy and sexual self-care in women: a cross-sectional study in Iran

**DOI:** 10.1093/sexmed/qfaf071

**Published:** 2025-11-19

**Authors:** Fatemeh Darban, Nastaran Heydarikhayat, Sahar Olyaee, Nazanin Raeisi

**Affiliations:** Department of Nursing, School of Medicine, Iranshahr University of Medical Sciences, Iranshahr, 9818954579, Iran; Department of Nursing, School of Medicine, Iranshahr University of Medical Sciences, Iranshahr, 9818954579, Iran; Department of nursing, school of medicine, Student Research Committee, Iranshahr University of Medical Sciences, Iranshahr, 9818954579, Iran; Department of nursing, school of medicine, Student Research Committee, Iranshahr University of Medical Sciences, Iranshahr, 9818954579, Iran

**Keywords:** sexual health literacy, sexual self-care, self-care model, women’s health, reproductive health, health behavior

## Abstract

**Background:**

Sexual self-care behaviors in women play a crucial role in promoting their sexual health, ultimately enhancing family and societal well-being. Adequate knowledge and the ability to apply such information are key factors influencing sexual self-care.

**Aim:**

This study was conducted to examine the relationship between sexual health literacy and sexual self-care among women.

**Methods:**

This cross-sectional study was conducted among 435 women of reproductive age attending healthcare centers affiliated with Iranshahr University of Medical Sciences, Iran, during 2024-2025. Data were collected using validated questionnaires on sexual self-care and the Iranian Adult Sexual Health Literacy scale. Statistical analysis was performed using SPSS 22, employing logistic regression, analysis of variance (ANOVA), and multiple linear regression models.

**Outcomes:**

Determined the relationship between sexual health literacy and sexual self-care in young women.

**Results:**

The results revealed a statistically significant direct association between sexual self-care and sexual health literacy (*P* = .000). Women demonstrating higher sexual health literacy scores reported higher sexual self-care scores. Sexual self-care was associated with total sexual health literacy scores, as well as with the dimensions of information access skills and information application skills.

**Clinical Implications:**

The results provide a rationale for policymakers to implement targeted sexual health literacy programs, which can subsequently enhance self-care practices.

**Strengths and Limitations:**

The strengths of this study include the use of robust statistical methods and a large, representative sample size. However, limitations should be noted, such as the dependence on self-reported data, which may introduce bias, and the restricted generalizability of findings due to the single-region, cross-sectional study design.

**Conclusion:**

This study demonstrated that improved sexual health literacy enhances sexual self-care scores in women. These results highlight the crucial need to enhance women’s sexual health literacy.

## Introduction

In developing countries with shortage of trained healthcare providers and inadequate medical infrastructure, sexual self-care can serve as an effective strategy for protecting women’s health and preventing diseases.^1^ Sexual self-care interventions encompass diverse approaches, including self-screening for sexually transmitted infections (STIs), HIV, and pregnancy, as well as contraceptive options such as condoms, over-the-counter oral contraceptives (OCs), and emergency contraceptive pills.^2^ These sexual self-care practices empower women and girls with essential knowledge and skills to navigate reproductive health processes—including menstruation health, fertility management, pregnancy, and childbirth—while also equipping them to provide essential newborn and child care.^3^ Sexual self-care confers significant physical, emotional, and mental health benefits. This multidimensional practice encompasses maintaining safe sexual behaviors, obtaining routine sexual health screenings, and fostering bodily awareness and sexual autonomy through self-exploration.^4^ Moreover, these practices enable healthcare providers to detect ineffective and high-risk behaviors, informing the design of targeted educational initiatives and evidence-based clinical interventions.^5^

Individuals may adopt sexual self-care practices due to their convenience, cost-effectiveness, empowerment, and better alignment with personal values, cultural norms, and lifestyles. In contexts where discrimination and inequality prevail, self-care often becomes an essential means of bypassing formal healthcare systems, allowing individuals to overcome systemic barriers such as stigmatizing provider interactions, privacy concerns, poor service quality, and limited access to care.^6-8^ Sexual motivations and behaviors are profoundly influenced by context, ethnic, racial, and cultural factors.^1^ Concurrently, promoting responsible sexual behavior and safeguarding reproductive health continue to pose substantial challenges among adolescents and young adults, constituting critical societal priorities worldwide.^9^

Results from various studies indicate that unmet sexual needs, often resulting from lack of skills, information, or awareness, constitute key factors in sexual dysfunction.^10^^,^^11^ Sexual health literacy, conceptualized as a specialized domain of general health literacy, is defined as “a set of knowledge, attitudes, beliefs, motivations, and personal competencies that enable individuals to access, understand, evaluate, and utilize sexual and reproductive health information in daily life, facilitating discourse, judgment, and decision-making to address sexual concerns while enhancing sexual health, relationships, and overall wellbeing.”^12^^,^^13^ Multiple studies have demonstrated that sexual health literacy influences sexual functioning.^14-16^

Sexual health literacy enhances individuals’ capacity to effectively utilize health information and services, while simultaneously shaping sexual knowledge, attitudes, and beliefs—collectively improving sexual satisfaction.^13^^,^^17^ Sexual health literacy serves as a critical psychosocial asset that enhances informed sexual decision-making, confers protection against adverse sexual health outcomes, including STIs and reproductive health complications, and fosters constructive dialogue about sexual health matters in both personal and clinical contexts. This multidimensional competency emerges as a fundamental contributor to optimal sexual health and holistic well-being.^16^

Sexual and reproductive self-care among Iranian women and adolescent girls represents a multidimensional concept shaped by individual-family interactions, perceptions of premarital sexual relations, enabling factors for sexual/reproductive self-care, understanding of behaviors and interactions with the opposite sex, communication barriers with parents, and knowledge of sexual/reproductive health. In Iran, cultural, historical, social, legal, and religious considerations have made sexual health issues a taboo subject, resulting in limited attention to this area.^18^ Consequently, sexual health research has consistently faced challenges, with most existing studies focusing primarily on high-risk sexual behaviors among adolescents and youth.^4^^,^^19^^,^^20^ Despite conducting an extensive literature review, no studies were found examining the relationship between sexual health literacy and women’s sexual self-care behaviors. This critical research gap raises a fundamental question: Is there a significant association between sexual health literacy and sexual self-care practices among women in southeastern Iran?

## Methods

### Participants

This correlational cross-sectional study was conducted from July 2024 to April 2025 in community health service centers affiliated with Iranshahr county in southeastern Iran. The study involved young women receiving services from comprehensive health centers in Iranshahr province. A stratified random sampling method was employed, with urban health centers assigned to one stratum and rural health centers to another. Participants were randomly selected from both strata: urban and rural. The sample size was determined using Morgan’s table, yielding 380 participants. To account for potential attrition, a final sample size of 435 was considered. The inclusion criteria were as follows: being married, residing in Iranshahr province, aged under 40 years, and possessing literacy skills or the ability to communicate verbally and auditorily. Incomplete questionnaires were excluded from the analysis. Following approval by the University Research Council and ethical committee, and after obtaining official permissions, the research team initiated stratified sampling. The research team obtained a complete list of eligible young women accessing services through Iranshahr province’s healthcare system, categorized by urban and rural residency. Through computer-generated random sampling, 435 participants were enrolled (urban: n = 272, rural: n = 163).

### Data collection methods

The research team conducted initial recruitment via telephone interviews, wherein researchers formally introduced their institutional affiliation and comprehensively outlined the study’s objectives and protocols to potential participants. After obtaining consent and agreeing on the most convenient location for questionnaire completion, data collection was conducted either at health centers (n = 300) or participants’ homes (n = 135). Researchers provided detailed explanations about the study procedures and questionnaire completion, remaining present throughout the process to address any questions or clarify ambiguities. All the questionnaires were collected immediately after completion by the researchers themselves. Participants were assured of complete confidentiality. The average completion time for questionnaires was approximately 20 minutes.

### Measures

The demographic data form included participants’ age, marital status, number of children, occupation, place of residence (urban/rural), age at marriage, age at first childbirth, spouse’s occupation, and spouse’s education level, along with other relevant sociodemographic characteristics.

#### Sexual self-care questionnaire (FSHS)

The Persian version of the Female Sexual Health Self-care (FSHS) questionnaire was developed by Yazdani et al. (2022) for women of reproductive age.^21^ This 40-item instrument assesses 4 domains: (1) prevention of STIs, (2) prevention of gynecological cancers, (3) prevention of unintended pregnancies, and (4) promotion of sexual health. Responses are scored on a 5-point Likert scale ranging from “never”^1^ to “always.”^5^ In order to better understand scoring and its comparability, the scores of each factor were converted into 0-100 scores, and as a result, the score of the instrument was considered 0-100. Scores from 0 to 50 are considered inadequate health literacy, 50.1 up to 75 are considered insufficient health literacy, and 75.1 to 100 are considered adequate sexual self-care. The tool’s validity was established through content validity analysis, yielding excellent Content Validity Index (CVI) (0.93) and Content Validity Ratio (CVR) (0.96) scores. Internal consistency reliability was confirmed with a Cronbach’s alpha coefficient of 0.94. In a separate reliability assessment involving 30 mothers, the questionnaire demonstrated strong consistency (*α* = 0.91).^21^

#### Sexual health literacy

The sexual health literacy data were collected using the validated Sexual Health Literacy for Adults (SHELA) questionnaire developed by Masoumi et al. (2019), which consists of 40 items measuring 4 key dimensions: access to information, reading and comprehension, evaluation and analysis, and application of health information. Engaging with this field effectively requires access to specialized databases and credible sources. Individuals with adequate literacy skills must critically analyze the available materials, identify relevant resources for deeper examination, and ultimately evaluate their suitability for application through rigorous assessment. Responses were scored on a 5-point Likert scale ranging from 1 (strongly disagree) to 5 (strongly agree).

The instrument’s raw scores were transformed to a standardized scale ranging from 0 to 100, with established cut-points defining three distinct health literacy categories: inadequate (0-50), insufficient (50.1-66), and adequate (66.1-100). Psychometric evaluation confirmed strong content validity (CVR 0.84, CVI 0.81) and high reliability: access (*α* = 0.94), reading/comprehension (*α* = 0.98), evaluation/analysis (*α* = 0.77), and application (*α* = 0.94), with outstanding full-scale consistency (*α* = 0.98).^22^

### Data analysis

The data were analyzed using SPSS software (version 23). Descriptive statistics, including mean, standard deviation, and frequency distribution tables (absolute and relative), were used to summarize the demographic characteristics. The Kolmogorov-Smirnov test results indicated that quantitative variables in this study followed a normal distribution. Therefore, parametric statistical methods were used for comparative analysis of the variables. The relationship between sexual self-care and sexual health literacy was examined using Pearson’s correlation coefficient and linear regression analysis. One-way ANOVA was employed to assess the association between women’s demographic characteristics and different levels of sexual self-care scores. All the analyses were performed with a 95% confidence interval and a significance level set at *α* = .05.

## Results

### Demographic characteristics

The participants had a mean age of 33.6 ± 9.1 years (range: 16-40 years), with the majority being married (96.6%). The mean age at marriage was 19.1 ± 5.3 years (range: 15-39 years), while the mean monthly household income was $100 (equivalent to 80 million Iranian rials, the national currency). More details are provided in Table 1.

**Table 1:** Demographic characteristics of participants.

**Variables**		**N (%)**	**Total (N)**
**Age (mean ± SD)**	9.1 ± 33.6		435
**Age at marriage (mean ± SD)**	5.3 ± 19.1		435
**Average age of first child (mean ± SD)**	7.8 ± 9.7		435
**Marital status**	Married	420 (96.6)	435
	Divorced	11 (2.5)	
	Widowed	4 (0.9)	
**Living place**	Urban	272 (62.5)	435
	Rural	163 (37.5)	
**Education level**	Primary or less	47 (10.8)	435
	Middle school	158 (36.3)	
	High school	137 (31.5)	
	University degree	93 (21.4)	
**Occupational status**	Homemaker	360 (82.8)	435
	Part-time employment	57 (13.1)	
	Full-time employment	18 (4.1)	
**Number of children**	2≥	282 (64.8)	435
	4-3	104 (23.9)	
	6-5	41(9.4)	
	≤7	8 (1.8)	
	Inadequate (0-50)	86 (19.9)	435
**Levels of sexual health literacy**	Fairly adequate (50.1-66)	132 (30.2)	
	Adequate (66.1-100)	217 (49.9)	
	Inadequate (0-50)	196 (45.0)	435
**Levels of sexual self-care**	Fairly adequate (50.1-75)	223 (51.2)	
	Adequate (75.1-100)	16 (3.8)	

### Sexual self-care

The study revealed a mean sexual self-care score of 15.6 ± 58.0 (possible range: 40-89.5), with 45% of mothers demonstrating inadequate sexual self-care practices and 51.2% showing moderately adequate levels, a noteworthy finding. Analysis of specific domains showed the lowest scores in cancer and pregnancy prevention, while the highest scores were observed in sexual health promotion and STI prevention (Table 2). Notably, optimal levels were not attained in any sexual self-care domain, with significant proportions of mothers demonstrating suboptimal practices: 48.3% in STI prevention, 63.1% in cancer prevention, 45.8% in pregnancy prevention, and 35.3% in sexual health promotion (Table 3).

**Table 2:** Mean scores of sexual self-care and sexual/reproductive health literacy dimensions among women (N: 435).

**Variables**	**SD ± mean**	**Minimum**	**Maximum**
**Sexual self-care**	15.6 ± 58.0	20.0	89.5
**Prevention of STIs and genital tract infections**	20.1 ± 60.5	20.0	98.6
			
**Prevention of gynecological cancers**	22.5 ± 46.7	20.0	100.0
**Prevention of unintended pregnancy**	16.9 ± 52.8	20.0	100.0
**Promotion of sexual health**	17.9 ± 63.8	20.0	100.0
**Sexual and reproductive health literacy**	20.6 ± 70.8	20.0	100.0
**Skills of access of information**	28.8 ± 63.2	20.0	100.0
**Skills of reading and understanding information**	42.2 ± 72.2	20.0	100.0
**Skills of evaluation and analysis of information**	21.7 ± 71.8	20.0	100.0
**Skills of application of information**	16.9 ± 74.1	20.000	100.0

Abbreviation: STI, sexually transmitted infection.

**Table 3:** Correlation of sexual self-care and sexual health literacy.

**Variable**	**Sexual self-care**	
**Sexual health literacy and dimensions**	**Pearson correlation**	***P*-value**
**Sexual and reproductive health literacy**	0.724	.000
**Skills of access of information**	0.696	.000
**Skills of reading and understanding information**	0.632	.000
**Skills of evaluation and analysis of information**	0.575	.000
**Skills of application of information**	0.658	.000

### Sexual health literacy

The women’s mean sexual health literacy score was 20.6 ± 70.8 (possible range: 20-100), with 49.9% demonstrating adequate sexual health literacy levels and 19.9% displaying inadequate proficiency. Analysis of specific dimensions revealed the highest scores in information application skills, followed by comprehension skills and analysis/evaluation skills, while the lowest scores were observed in information access skills (Table 2). Notably, the proportion of mothers with adequate skills varied across dimensions: 40.8% in information access, 54.2% in information comprehension, 54.0% in information analysis/evaluation, and 49.9% in information application (Table 3).

### The relationship between sexual self-care and sexual health literacy

Pearson correlation analysis demonstrated significant positive relationship between sexual self-care scores (including all dimensions) and sexual health literacy scores (with all components) (*P* < .001). Higher sexual health literacy scores consistently predicted elevated sexual self-care scores across all measured dimensions.

Linear regression analysis indicated that women’s sexual health literacy scores are associated with their sexual self-care. Specifically, variations in women’s’ sexual health literacy scores explained 52.4% of the variance in their sexual self-care scores. The findings demonstrated that for every 1-unit increase in women’s sexual health literacy scores, their sexual self-care scores increased by 0.55 points.

Furthermore, linear regression results showed that the dimensions of “information access skills” and “information application skills” in sexual health literacy were significant predictors of women’s sexual self-care. Each 1-unit increment in these dimensions was associated with a 57.3% increase in sexual self-care scores (*β* = .573, *P* < .001). Each additional point in information access skills (*β* = .241) and application skills (*β* = .318) significantly predicted higher sexual self-care scores (both *P* < .001) (Table 4).

**Table 4:** Linear regression analysis of sexual health literacy dimensions as predictors of sexual self-care.

**Dependent variables**	***B***	**SE**	**Beta**	***T***	***P***	**Model summary**
**Constant** **Sexual and reproductive health literacy**	19.289 0.547	1.849 0.025	- .724	10.430 21.797	.000 .000	*R* = 0.724 *R*^2^ = 0.524 ADJ.R^2^ = 0.523
**Constant**	17.097	2.307	–	7.412	.000	*R* = 0.757 *R*^2^ = 0.573 ADJ.R^2^ = 0.569
**Skills of access of information**	0.241	0.31	.449	7.874	.000	
**Skills of reading and understanding of information**	0.022	0.041	.035	0.549	.583	
**Skills of evaluation and analysis of information**	0.011	0.038	.016	0.293	.769	
**Skills of application of information**	0.318	0.043	.346	7.305	.000	

## Discussion

This cross-sectional study investigated the relationship between sexual health literacy and self-care practices in a population of young women, uncovering strikingly low rates of optimal sexual self-care (3.8%). Nearly half of the women (49%) exhibited desirable sexual health literacy. A strong positive correlation was observed between literacy levels and sexual self-care practices, with sexual health literacy significantly predicting sexual self-care.

The mean sexual self-care score was 58.0 ± 15.6, indicating that 96.2% of the participants failed to meet optimal practice standards, demonstrating either moderate or inadequate self-care. Yazdani et al. (2023) reported a significantly higher mean sexual self-care score (70.66 ± 12.52), likely due to their participants’ higher educational attainment (minimum high school diploma vs 47% with middle school or less in our study).^21^ Yazdani et al.’s study was conducted in a major urban center in northern Iran, potentially affording participants greater access to reproductive health information and resources compared to rural populations. Geographical differences may also play a role, as our study included 37.5% rural participants from southeastern Iran. The researcher noted that, as in many traditional societies, sexual discourse among the Baloch people remains highly stigmatized, where open discussions are considered taboo and often associated with profound shame.^23^ This deeply entrenched cultural stigma consequently serves as a significant barrier to participation in sexual health initiatives aimed at improving self-care practices.

Regarding sexual health literacy, 49.4% of the mothers in the current study demonstrated adequate levels. This finding aligns with a study conducted in Qazvin by Panahi et al., which reported that 50.1% of the participants had sufficient sexual health literacy.^24^ However, it contrasts with studies by Vongxay et al. (2019) and Dabiri et al. (2019), where participants exhibited inadequate sexual health literacy levels.^25^^,^^26^

Findings from a UK study indicated that approximately 72% of the participants had adequate health literacy.^27^ In contrast, a study of African-American adults revealed that 65% exhibited low or inadequate health literacy levels.^28^ These disparities likely reflect variations in socioeconomic and cultural contexts across study populations. Furthermore, racial, ethnic, and cultural variations in health expectations may contribute to the observed heterogeneity in outcomes. Additionally, methodological differences across studies, including variations in measurement instruments and gender distribution among participants, could account for the inconsistent findings.

Our analysis of sexual health literacy dimensions revealed that participants scored lowest in the information access domain, aligning with Dabiri et al.’s (2019) findings.^26^ This contrasts with Banaei et al.’s (2025) results, which identified the evaluation and analysis dimension as the most challenging area.^13^ This variation likely stems from population-level disparities in access to sexual health information. In particular, restrictive cultural environments—especially for women—create multiple barriers to acquiring accurate sexual health knowledge. These barriers include limited parental education, inadequate school-based sexuality education, pervasive social taboos, insufficient healthcare provider awareness, and deeply entrenched norms of sexual shame.

The study findings revealed a statistically significant positive correlation between sexual self-care and sexual health literacy among women, suggesting that higher levels of sexual health literacy were associated with more comprehensive sexual self-care behaviors. While no prior studies were identified that explicitly examined the relationship between sexual self-care and sexual health literacy despite a comprehensive search of multiple databases, the current findings align with broader evidence linking sexual health literacy to improved sexual function outcomes.

Studies by Banaei et al. (2025) and Dehghankar et al. (2021) demonstrated that sexual health literacy has a direct and positive impact on sexual function.^13^^,^^15^ As mothers’ sexual health literacy improves, they demonstrate a greater capacity to translate this knowledge into practical strategies that optimize both physical and psychological aspects of sexual well-being. This enhanced understanding is associated with improved sexual function and may facilitate more effective sexual self-care practices.^11^^,^^29^

Sexual health literacy is a cognitive variable^30^ that influences behavior, as established by the well-documented relationship between knowledge and behavioral outcomes.^31^^,^^32^ This evidence suggests that sexual health literacy can significantly impact both sexual behavior and sexual self-care practices.^33^ Several studies have demonstrated that developing strong health literacy beliefs enhances individuals’ ability to perform health-related behaviors effectively.^34-36^ Individuals who are competent and confident in their health literacy skills are more likely to apply these skills to solve health-related problems.^35^ Similarly, sexual health literacy has been shown to promote healthier sexual relationships and behaviors through enhancing its positive effect on sexual self-efficacy.^13^ These findings align with theoretical models positing health literacy as both a facilitator and outcome of sexual self-efficacy, establishing a mutually reinforcing cycle that perpetuates positive health behaviors in sexual domains.

Another study demonstrated that educational interventions through school systems can empower adolescent girls, thereby improving their sexual and reproductive health status.^37^ Given women’s pivotal role in sexual and reproductive health self-care, comprehensive sexual education emerges as a fundamental determinant of positive sexual health outcomes.^38-41^ As key facilitators of self-care in sexual and reproductive health, maternal sexual education is strongly linked to increased sexual health literacy, reduced STI transmission, and strengthened interpersonal dynamics—essential elements of comprehensive sexual health promotion.^38^^,^^40^

## Conclusions

The primary novelty of this study is its use of standardized sexual health literacy scores to predict women’s sexual self-care behaviors. The study shows that women’s ability to access, comprehend, and implement sexual health knowledge is positively associated with enhanced sexual self-care practices. These results emphasize the necessity for comprehensive sexual health literacy interventions, including improved access to credible resources, targeted education on self-care practices, and enhanced provider training to address sexual health issues during clinical consultations. Moreover, the study advocates for the integration of routine sexual health evaluations into standard clinical practice to enhance women’s sexual well-being. By empirically linking health literacy to self-care behaviors, this research offers both a conceptual foundation and practical tools for designing more effective, culturally sensitive interventions to optimize women’s sexual health outcomes. These findings address an important gap in maternal health research and offer concrete directions for future public health initiatives and clinical practice improvements in this often-overlooked aspect of women’s health.

This study has several limitations. The primary limitation is the absence of prior research investigating the relationship between sexual health literacy and sexual self-care among women, highlighting a crucial knowledge gap that demands immediate scholarly attention. Additionally, dependence on self-reported measures may introduce recall and social desirability biases, potentially affecting data validity. Sociocultural stigma surrounding sexual health discourse may have constrained participants’ willingness to disclose sensitive information, potentially introducing reporting bias and affecting care quality. Additionally, as a cross-sectional study conducted in one region, the findings may lack generalizability given the known sociocultural influences on sexual health behaviors and attitudes. We strongly recommend replicating this research in diverse geographical locations with larger sample sizes to enhance the external validity of the results.

## Data Availability

The datasets generated during the current study are available from the corresponding author upon reasonable request.
